# Confinement of an alkaline environment for electrocatalytic CO_2_ reduction in acidic electrolytes†

**DOI:** 10.1039/d3sc01040f

**Published:** 2023-05-02

**Authors:** Xiaozhi Li, Peng Zhang, Lili Zhang, Gong Zhang, Hui Gao, Zifan Pang, Jia Yu, Chunlei Pei, Tuo Wang, Jinlong Gong

**Affiliations:** a School of Chemical Engineering & Technology, Key Laboratory for Green Chemical Technology of Ministry of Education, Tianjin University Tianjin 300072 China jlgong@tju.edu.cn; b Collaborative Innovation Center of Chemical Science & Engineering (Tianjin) Tianjin 300072 China; c Haihe Laboratory of Sustainable Chemical Transformations Tianjin 300192 China; d National Industry-Education Platform of Energy Storage, Tianjin University 135 Yaguan Road Tianjin 300350 China; e Joint School of National University of Singapore, Tianjin University International Campus of Tianjin University, Binhai New City Fuzhou 350207 China

## Abstract

Acidic electrochemical CO_2_ reduction reaction (CO_2_RR) can minimize carbonate formation and eliminate CO_2_ crossover, thereby improving long-term stability and enhancing single-pass carbon efficiency (SPCE). However, the kinetically favored hydrogen evolution reaction (HER) is generally predominant under acidic conditions. This paper describes the confinement of a local alkaline environment for efficient CO_2_RR in a strongly acidic electrolyte through the manipulation of mass transfer processes in well-designed hollow-structured Ag@C electrocatalysts. A high faradaic efficiency of over 95% at a current density of 300 mA cm^−2^ and an SPCE of 46.2% at a CO_2_ flow rate of 2 standard cubic centimeters per minute are achieved in the acidic electrolyte, with enhanced stability compared to that under alkaline conditions. Computational modeling results reveal that the unique structure of Ag@C could regulate the diffusion process of OH^−^ and H^+^, confining a high-pH local reaction environment for the promoted activity. This work presents a promising route to engineer the microenvironment through the regulation of mass transport that permits the CO_2_RR in acidic electrolytes with high performance.

## Abstract 2

This paper describes the design of hollow-structured Ag@C electrocatalysts to adjust the local reaction environment for high-performance CO_2_RR in acidic media.

## Graphical abstract

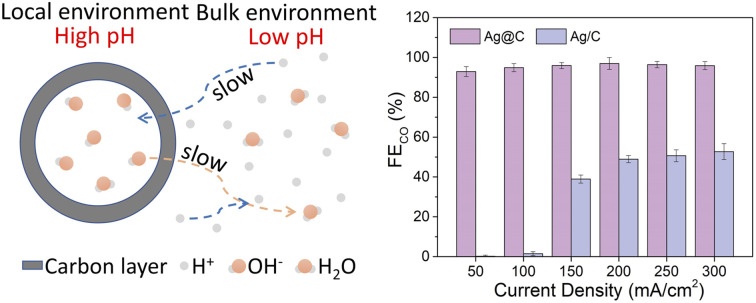

## Introduction

Converting CO_2_ into value-added chemical feedstocks and fuels through the electrochemical CO_2_ reduction reaction (CO_2_RR) driven by renewable electricity is an appealing pathway to develop a sustainable carbon cycle.^1–3^ In order to boost the catalytic activity, alkaline or neutral electrolysis systems are widely employed to suppress the competing hydrogen evolution reaction (HER), which could also promote CO_2_ activation and C–C coupling.^4–9^ However, the inevitable reaction between CO_2_ and local/bulk OH^−^ in alkaline or neutral electrolytes would lead to undesired CO_2_ consumption and crossover.^10–12^ This phenomenon results in low carbon utilization efficiencies (≤50% for C_1_ products and ≤ 25% for C_2+_ products) and limited energy efficiencies.^13–16^ Furthermore, the stability of alkaline CO_2_RR systems could be significantly lowered due to the accumulation of the generated carbonate.^13,17^ Therefore, it is essential to develop efficient CO_2_RR systems with high CO_2_ utilization efficiency and good stability.^18,19^

Among the emerging strategies to achieve this goal, acidic CO_2_RR has received considerable attention.^20,21^ Due to the high proton concentration and the use of a proton exchange membrane (PEM), the CO_2_RR in acidic media offers a viable approach to reduce the formation of carbonate and eliminate CO_2_ crossover.^22^ Therefore, acidic CO_2_RR can break the theoretical limitation of single-pass carbon efficiency (SPCE) for alkaline CO_2_RR with less energy cost for product separation.^23,24^ Without the accumulation of carbonate, long-term stability can also be improved in acidic systems. Furthermore, higher conductivity and the economic feasibility of PEMs also make it promising for industrialization.^16,25^ However, the kinetically favored HER is normally predominant in acidic electrolytes, which leads to a low faradaic efficiency (FE) of CO_2_RR products.^15,26^ Therefore, it is imperative to suppress the severe HER to improve the practical viability of acidic CO_2_RR.

The suppression of the HER in acidic media can be realized by the design of appropriate electrocatalysts. Bimetallic catalysts have been proven to modulate CO* coverage and weaken H* adsorption through adsorbate–adsorbate interactions, thus inhibiting the HER.^27^ Meanwhile, the CO_2_RR can be further enhanced by modifying the catalyst surface with basic sites to promote CO_2_ adsorption.^20,28^ Besides the design of novel catalysts, the optimization of the microenvironment is equally critical to render acidic CO_2_RR feasible.^29–34^ For instance, a hydrophobic chemical environment has been proven to benefit the CO_2_RR due to the high local CO_2_/H_2_O ratio in the gas diffusion electrode (GDE).^23,35^ It can be realized by adding polytetrafluoroethylene nanoparticles into the catalyst layer. According to recent studies, the H^+^ coverage close to the cathode surface was sensitive to the K^+^ concentration due to the competitive adsorption of cations at the outer Helmholtz plane (OHP), and thus concentrating potassium cations in the vicinity of active sites could promote the performance of the CO_2_RR.^16,24,36,37^ Besides, controlling the formation rate of CO/OH^−^ to compensate for the diffusion of protons from the bulk electrolyte and coating the catalyst layer with a nanoporous ion-regulatory layer to suppress the diffusion of K^+^ and OH^−^ are effective ways to lower the concentration of protons near the catalyst surface.^38,39^ In these cases, the competitive HER from proton reduction can be effectively suppressed. Meanwhile, the crossover of CO_2_ is minimized because any locally generated carbonate can be converted back to CO_2_ by the adequate protons in the bulk acidic electrolyte.^40^ Overall, engineering the local reaction environment through suitable catalyst design is essential for promoting acidic CO_2_RR.

In this study, Ag@C electrocatalyst with Ag active sites loaded on the interior surface of hollow carbon spheres was designed, which exhibits a CO FE (FE_CO_) of over 95% even in a strongly acidic electrolyte (pH 1.1) with improved stability. An SPCE of 46.2% was achieved at a CO_2_ flow rate of 2 standard cubic centimeters per minute (sccm) in virtue of the inhibited carbonate formation in acidic CO_2_RR. Computational modeling demonstrated that the diffusion of OH^−^ through the hollow spheres was limited due to the presence of the porous carbon layer. Thus, the enrichment of OH^−^ in the nanochamber brought a local alkaline environment that can effectively suppress the HER. This work highlights the importance of mass transport manipulation in engineering the reaction microenvironment for high-performance CO_2_RR.

## Results and discussion

### Synthesis and characterization of electrocatalysts

**Fig. 1:**
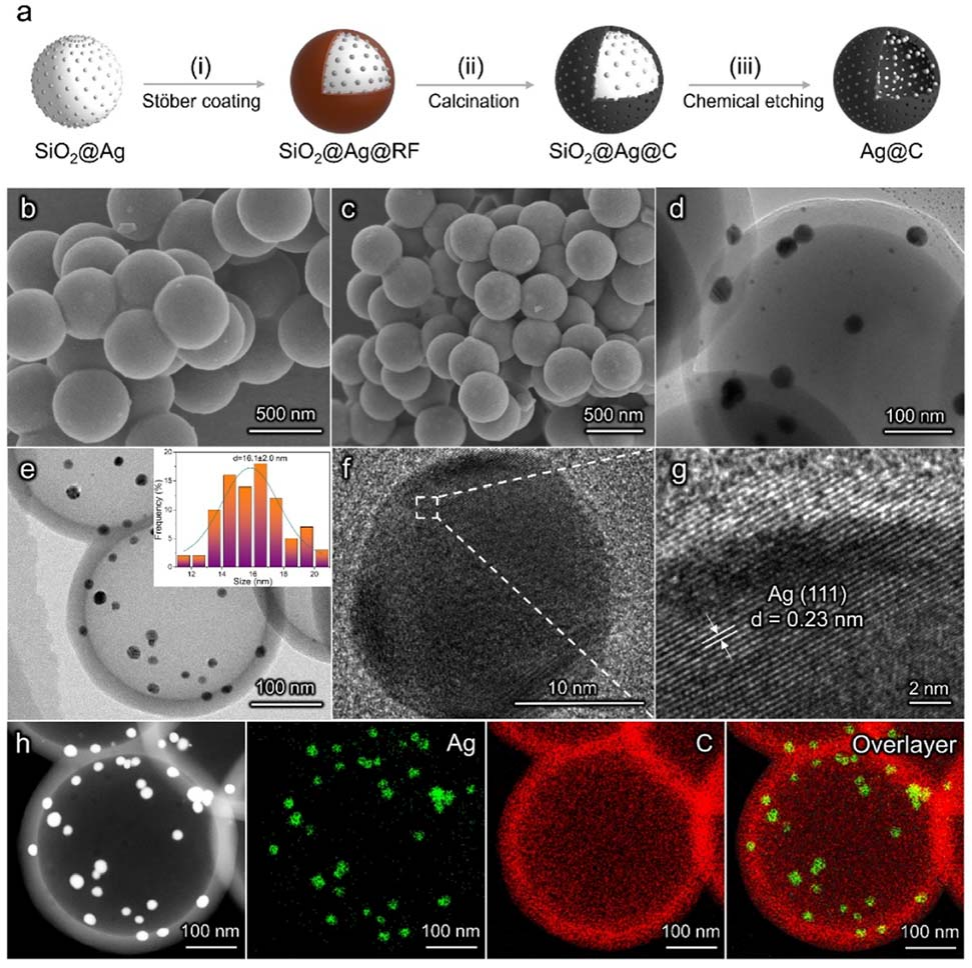
Schematic illustration and morphological characterization. (a) Scheme of the synthesis process of the Ag@C catalyst. (b) SEM image of SiO_2_@Ag@RF. (c) SEM and (d) TEM images of SiO_2_@Ag@C. (e) TEM and (f and g) HRTEM images of Ag@C. The inset in (e) is the particle size distribution of Ag. (h) Energy-dispersive spectroscopy (EDS) elemental mapping images of Ag@C.

The synthetic procedure of Ag@C catalysts is illustrated in Fig. 1a. First, SiO_2_ spheres with an average size of ∼400 nm were synthesized as templates (Fig. S1a†). Subsequently, Ag nanoparticles were loaded on the surface of SiO_2_ to obtain SiO_2_@Ag (Fig. S1b†).^41^ A modified Stöber coating method was adopted to fabricate SiO_2_@Ag@resorcinol-formaldehyde (SiO_2_@Ag@RF) spheres with a core–shell structure (Fig. 1b).^42^ Then, SiO_2_@Ag@C was obtained after a calcination process under an inert atmosphere, during which RF was converted to carbon at an elevated temperature. Scanning electron microscopy (SEM) and transmission electron microscopy (TEM) images of SiO_2_@Ag@C show the full coverage of the carbon shell without Ag particles left on the outer surface (Fig. 1c and d). The as-prepared SiO_2_@Ag@C was finally converted into Ag@C *via* a chemical etching treatment. TEM observation indicates the retention of a hollow spherical morphology with a shell thickness of ∼40 nm after the successful removal of the SiO_2_ cores. The Ag particles (average diameter ≈ 16.1 nm) are confined inside the carbon spheres (Fig. 1e). As shown in the high-resolution TEM (HRTEM) images, the lattice spacing of the Ag@C catalyst corresponds to the (111) facet of Ag (Fig. 1f and g). The elemental mapping images of Ag@C further reveal that Ag nanoparticles are located on the inner surface of the carbon shell (Fig. 1h). The structural feature of the carbon shell was studied using N_2_ adsorption–desorption measurements (Fig. S2†). The Ag@C catalyst shows a porous structure with an average pore size of about 2.6 nm, which ensures sufficient reactants near the surface of the active sites (Fig. S3†). Conventional Ag/C catalysts without a core–shell structure were synthesized as the control sample (Fig. S5†). X-ray diffraction (XRD) patterns indicate the presence of metallic Ag in Ag@C and Ag/C catalysts (Fig. S6†). X-ray photoelectron spectroscopy (XPS) measurements were performed to further confirm the chemical state of the Ag active sites (Fig. S7†). The Ag 3d_5/2_ peak at 368.2 eV was observed, implying that the metallic Ag is the active phase in both catalysts. Both Ag@C and Ag/C catalysts possess approximately the same mass loadings (∼40%) and similar Ag nanoparticle sizes (insets of Fig. 1e, insets of Fig. S5a and Table S1†). Meanwhile, the Raman intensity ratios between D and G bands in both catalysts are similar, which implies the consistent properties of the carbon supports (Fig. S8†).^42^ In addition, a C@Ag catalyst with active sites loaded on the outer surface of a porous carbon layer was prepared to further demonstrate the importance of the unique structure of the Ag@C catalyst in enhancing the acidic CO_2_RR performance (Fig. S9†). The preparation of C@Ag followed a similar synthesis procedure to the Ag@C catalyst.

### CO_2_ electroreduction in alkaline and acidic media

**Fig. 2:**
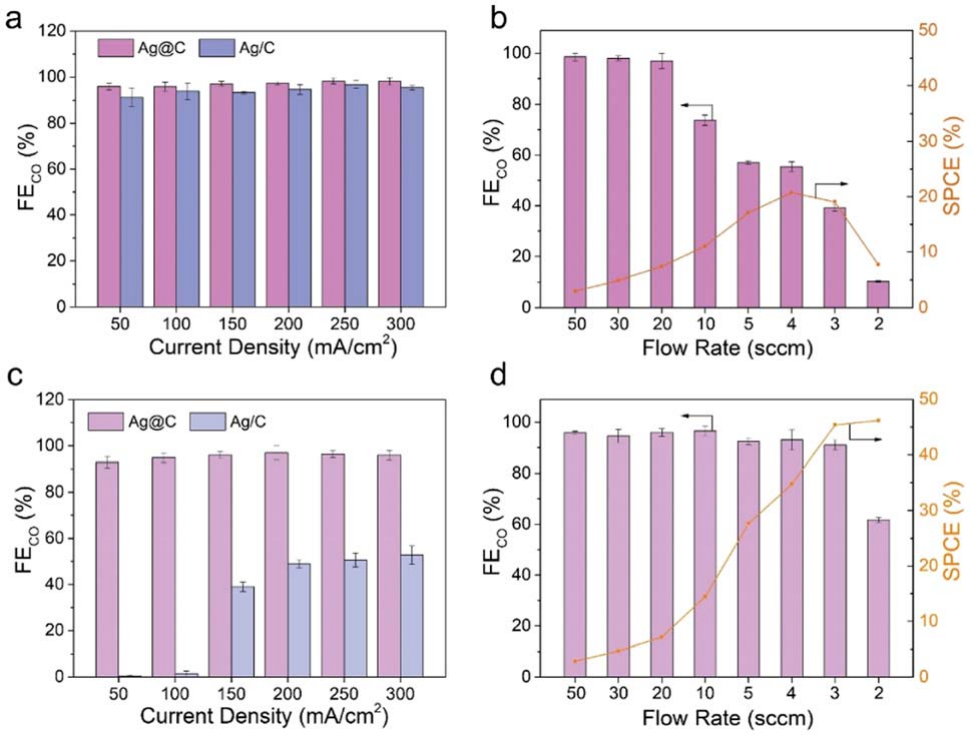
CO_2_RR performance in alkaline and acidic media. (a) FE_CO_ on Ag@C and Ag/C at different current densities in 1.0 M KOH (pH 13.7). (b) FE_CO_ and corresponding SPCE on Ag@C at 200 mA cm^−2^ with different CO_2_ flow rates in 1.0 M KOH. (c) FE_CO_ on Ag@C and Ag/C at different current densities in 0.5 M K_2_SO_4_ (pH 1.1). (d) FE_CO_ and corresponding SPCE on Ag@C at 200 mA cm^−2^ with different CO_2_ flow rates in 0.5 M K_2_SO_4_ (pH 1.1). Error bars represent the standard deviation from at least three independent measurements.

The CO_2_RR performance was evaluated in a typical three-electrode flow cell reactor. The flow rate of electrolyte was kept constant for all the tests. As shown in Fig. 2a, both Ag@C and Ag/C catalysts exhibited a similar FE_CO_ of over 90% in the current range of 50 to 300 mA cm^−2^ in the alkaline electrolyte (1.0 M KOH, pH 13.7), which is consistent with previous reports.^5,43,44^ This result shows that an alkaline environment is beneficial for the suppression of the HER. Besides, the catalytic activity of Ag@C was tested at different CO_2_ flow rates from 50 to 2 sccm in the alkaline electrolyte at 200 mA cm^−2^ (Fig. 2b). As the flow rate decreased, the FE_CO_ showed an apparent drop due to the rapid depletion of CO_2_ to CO_3_^2−^/HCO_3_^−^ (Fig. S10†). A relatively low maximum SPCE of 20.7% was achieved at 4 sccm.

As mentioned above, acidic CO_2_RR may offer a viable solution to improve the carbon utilization efficiency by minimizing the formation and crossover of carbonate. After the preliminary tests in mildly acidic media (Fig. S11†), the CO_2_RR was further performed under strongly acidic conditions to investigate the feasibility of the Ag@C catalyst (Fig. 2c and d). An acidified 0.5 M K_2_SO_4_ solution with a pH of around 1.1 was employed as the catholyte to ensure consistent cation concentrations in both acidic and alkaline electrolysis. Besides, a relatively high electrolyte concentration contributes to the reduction of cathodic ohmic loss (Fig. S12†). As shown in Fig. 2c, the Ag@C catalyst displayed a FE_CO_ of over 95% at a current density of 300 mA cm^−2^, which is comparable to the performance under alkaline conditions. Meanwhile, the activity of Ag@C significantly exceeded that of Ag/C and C@Ag catalysts over the whole tested current density range (Fig. S13†). More importantly, the Ag@C catalyst exhibited relatively high FE_CO_ even at low CO_2_ flow rates (Fig. 2d). A high SPCE of 46.2% was achieved at 2 sccm in the acidic media, implying the advantage in minimizing carbonate formation compared with the alkaline system.

**Fig. 3:**
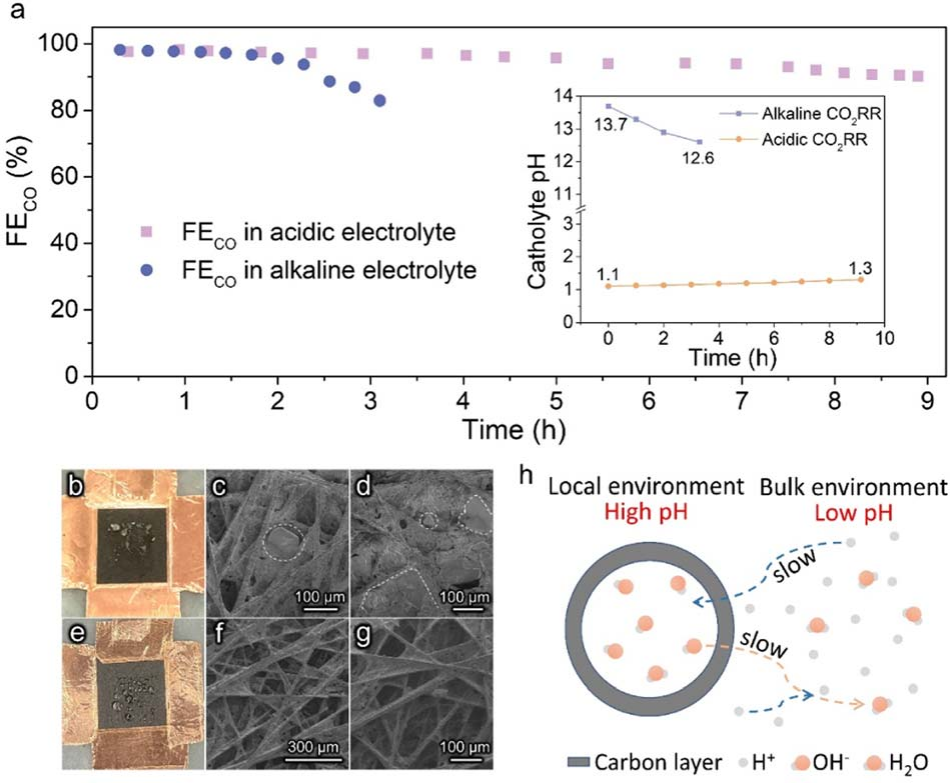
Stability performance of Ag@C in acidic and alkaline media. (a) Long-term performance of the CO_2_RR at 100 mA cm^−2^ in alkaline (1.0 M KOH, pH 13.7) and acidic (0.5 M K_2_SO_4_, pH 1.1) electrolytes. Inset shows the pH variation of the catholyte with time. (b) Photograph and (c and d) SEM images of the backside of the GDE after the stability test in 1.0 M KOH. (e) Photograph and (f and g) SEM images of the backside of the GDE after the stability test in 0.5 M K_2_SO_4_ (pH 1.1). (h) Schematic of the local reaction environment and ion transport on the Ag@C catalyst.

Stable operation is essential to ensure the CO_2_RR to be economically competitive for practical application. The stability of Ag@C in acidic and alkaline systems was examined under a constant current density of 100 mA cm^−2^ (Fig. 3a). An apparent deactivation of the alkaline CO_2_RR system was observed due to the precipitation of carbonate salt, as evidenced by the observation of the obvious salt on the backside of the gas diffusion electrode (GDE, Fig. 3b–d). In contrast, the FE_CO_ was maintained over 90% during ∼9 h of electrolysis in the acidic electrolyte. Post characterization studies demonstrate that the hollow structure of the Ag@C catalyst was well retained with negligible changes in composition (Fig. S14–S16†). The XPS data indicate the retention of metallic Ag after the long-term test (Fig. S17†), which excludes the effect of chemical state change of Ag active sites on the catalytic stability. Meanwhile, the outlet electrolyte was measured by inductively coupled plasma optical emission spectrometry (ICP-OES) after the durability test under acidic conditions. No dissolved Ag was detected (Table S2†). Therefore, the slight decline in FE_CO_ may result from the wetting of the gas diffusion layer (GDL) as suggested by Fig. 3e–g. In addition, only a minor shift in catholyte pH was observed after the long-term test in the acidic electrolyte (inset of Fig. 3a), which indicates that the stability of the electrolyte environment may also facilitate the stable operation of the whole electrolysis system. Furthermore, the stability of Ag/C in 0.5 M K_2_SO_4_ (pH 1.1) was also measured. The gradual increase of FE_H_2__ due to the severe flooding was also observed in the Ag/C catalyst (Fig. S18a†). And the TEM image after the long-term test shows no significant change in the catalyst morphology and the particle size distribution of Ag (Fig. S18b†), which reinforces the evidence that the wetting of GDL is responsible for the degradation of acidic electrolysis.

The relatively good performance of the Ag@C catalyst in acidic electrolysis may be attributed to the rational design of its structure. As shown in Fig. 3h, it is presumed that the presence of the carbon layer confines the CO_2_RR within the nanochambers and provides an obstacle to the diffusion process. In the course of the CO_2_RR, the diffusion of H^+^ from the bulk electrolyte is restricted by the carbon shell. Meanwhile, OH^−^ generated along with the reaction cannot diffuse out promptly as well. Thereby, the acidic electrolyte can be neutralized rapidly owing to the depletion of H^+^ and accumulation of OH^−^. As the reaction proceeds, the concentration gradient of OH^−^ inside and outside the carbon layer gradually increases, which means a higher diffusion flux. When the diffusion and formation rates of OH^−^ reach a dynamic equilibrium, a high internal concentration of OH^−^ remains approximately constant. Ultimately, an alkaline local environment is maintained near the surface of active sites to inhibit the HER effectively.

### Mass transport simulation

**Fig. 4:**
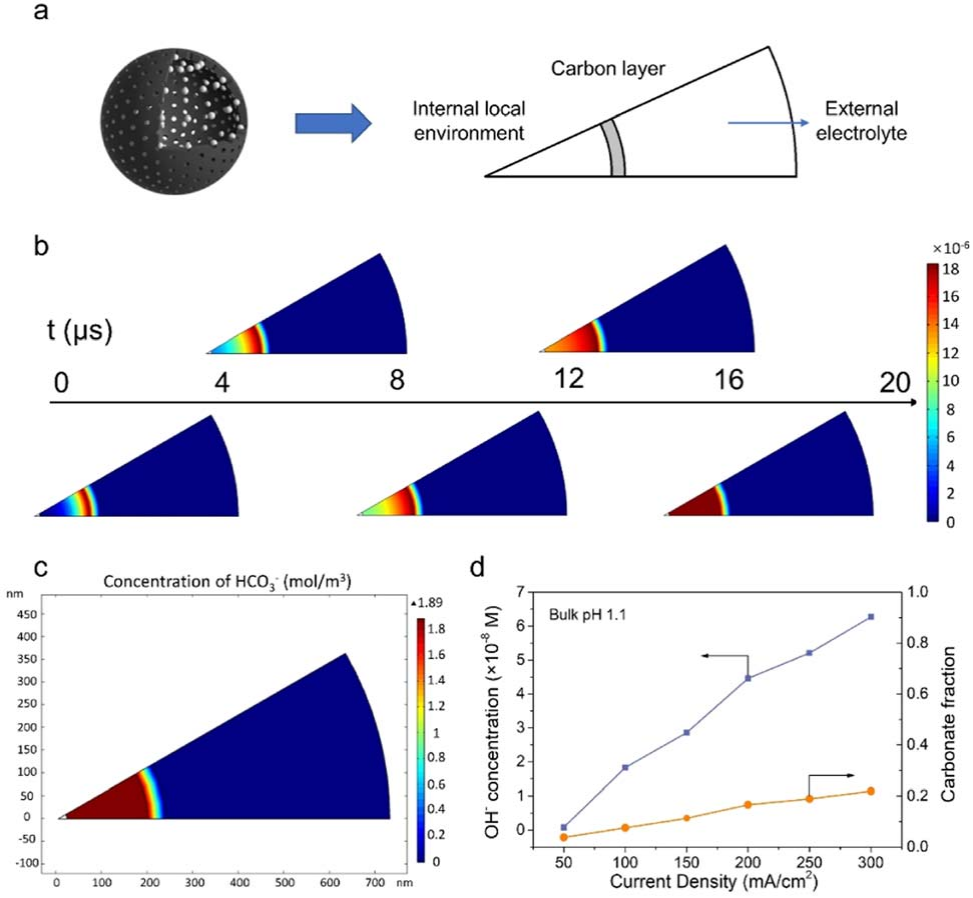
Mass transport simulations. (a) Graphical illustration of the modeling domains. (b) Variation of OH^−^ concentration with time. (c) HCO_3_^−^ concentration profile at the steady state with a current density of 100 mA cm^−2^. (d) OH^−^ concentration profile and carbonate fraction at different current densities with bulk pH 1.1. The carbonate fraction is calculated using the ratio between carbonate (HCO_3_^−^, CO_3_^2−^) and the sum of carbon species (HCO_3_^−^, CO_3_^2−^ and CO_2,aq_).

To further verify the proposed mechanism, mass transport simulation was adopted to explore the diffusion process in the acidic electrolyte. A sector domain was selected as the model for the calculation (Fig. 4a and S19†). The dimensions of the sector were defined according to the characterization results of SEM and TEM. In the simulations, CO_2_ molecules diffused to the cavity and converted into CO on the inner surface of the carbon shell.

As presented in Fig. 4b, the transient model shows the variation of OH^−^ concentration distribution over time (Movie S1†). It is clear that the carbon layer significantly restricts the outflow of locally generated OH^−^, which leads to a relatively high OH^−^ concentration inside the hollow sphere. Therefore, the high local pH could lead to an improved CO_2_RR performance, which is consistent with our hypothesis and experimental results. The distribution of carbonate was also assessed due to its potential impact on the system stability. An enriched carbonate concentration at the steady state was observed inside the cavity (Fig. 4c). With the increase of current density, a higher OH^−^ concentration was obtained and consequently led to an elevated carbonate fraction (Fig. 4d and S20–S25†). However, the carbonate ratio merely stayed at a relatively low value of 0.2 even at 300 mA cm^−2^, in agreement with the good stability of the CO_2_RR. Altogether, the mass transport simulation validates the impact of the catalyst structure on tuning the local environment and promoting the CO_2_RR performance in the acidic electrolyte.

## Conclusions

To summarize, acidic CO_2_RR offers a promising route to address the issues of relatively low carbon utilization efficiency and poor stability due to the inevitable formation and accumulation of carbonate in conventional alkaline systems. In this work, a stable CO_2_RR in the acidic electrolyte (pH 1.1) was realized without the compromise on FE_CO_ by the design of Ag@C catalysts. A high SPCE of 46.2% was achieved, which is approximately twice that of alkaline electrolysis. Mass transport simulation demonstrates that the structure of Ag@C plays a key role in regulating the diffusion pathways of H^+^ and OH^−^, consequently influencing the local pH at the surface of active sites. As a result, the confinement of an alkaline environment effectively inhibits the HER in the bulk acidic electrolyte for enhanced CO_2_RR performance. Overall, these findings suggest a general principle in the design of catalysts with controllable mass transport properties and emphasize the importance of microenvironment engineering for the CO_2_RR in acidic media.

## Data availability

The data that supports the findings of this study is available from the corresponding author upon reasonable request.

## Author contributions

J. L. G. supervised the project. P. Z. and X. Z. L. conceived the idea. X. Z. L. synthesized catalysts and conducted the CO_2_RR performance tests. L. L. Z. and Z. F. P. conducted mass transport simulations. G. Z., H. G., and J. Y. conducted related characterization. All the authors participated in the writing of the manuscript.

## Conflicts of interest

There are no conflicts to declare.

## Supplementary Material

SC-014-D3SC01040F-s001
