# Supplementary material for: Confinement of an alkaline environment for electrocatalytic CO2 reduction in acidic electrolytes
Source: Chem Sci. 2023 May 2;14(21):5602–7. doi: 10.1039/d3sc01040f (PMC10231308; doi:10.1039/d3sc01040f)
Supplement: SC-014-D3SC01040F-s001 [file SC-014-D3SC01040F-s001.pdf]

Electronic Supplementary Material (ESI) for Chemical Science.
This journal is © The Royal Society of Chemistry 2023

Supporting Information

Confinement of Alkaline Environment for Electrocatalytic CO_2 Reduction in Acidic Electrolyte

Xiaozhi Li[#,a,b], Peng Zhang[#,a,b,d,e], Lili Zhang[a,b], Gong Zhang[a,b], Hui Gao[a,b], Zifan Pang[a,b], Jia Yu[a,b], Chunlei Pei[a,b], Tuo Wang[a,b,c,d], and Jinlong Gong[a,b,c,d,*]

[a] *X. Li, Prof. Dr. P. Zhang, L. Zhang, G. Zhang, H. Gao, Z. Pang, J. Yu, Prof. Dr. C. Pei, Prof. Dr. T. Wang, Prof. Dr. J. Gong*

School of Chemical Engineering and Technology; Key Laboratory for Green Chemical Technology of Ministry of Education, Tianjin University, Tianjin 300072, China.

[b] *X. Li, Prof. Dr. P. Zhang, L. Zhang, G. Zhang, H. Gao, Z. Pang, J. Yu, Prof. Dr. C. Pei, Prof. Dr. T. Wang, Prof. Dr. J. Gong*

Collaborative Innovation Center of Chemical Science and Engineering (Tianjin), Tianjin 300072, China.

[c] *Prof. Dr. T. Wang, Prof. Dr. J. Gong*

Haihe Laboratory of Sustainable Chemical Transformations, Tianjin 300192, China.

[d] *Prof. Dr. P. Zhang, Prof. Dr. T. Wang, Prof. Dr. J. Gong*

National Industry-Education Platform of Energy Storage, Tianjin University, 135 Yaguan Road, Tianjin 300350, China.

[e] *Prof. Dr. P. Zhang*

Joint School of National University of Singapore and Tianjin University, International Campus of Tianjin University, Binhai New City, Fuzhou 350207, China.

# These authors contributed equally to this work.

*Corresponding author: jlgong@tju.edu.cn

Experimental Section

Chemicals and Materials

Tetraethyl orthosilicate (TEOS, 98%) was purchased from Tianjin Luoen Chemical Reagent Co., Ltd. Ethanol (HPLC, 99.8%), resorcinol (AR, 99%), polyvinylpyrrolidone (PVP, MW = 40000, K30), NaOH (AR, 96%), KOH (GR, 95%), K_2SO_4 (AR, 99%), $AgNO_3$ (AR, 99.8%) were purchased from Sigma-Aladdin. Formaldehyde solution (38%), H_2SO_4 (GR), $NH_3·H_2O$ (AR) were purchased from Tianjin Yuanli Technology Development Co., Ltd. FAA-3-SOLUT-10 (10 wt%), FAA-3-50 membrane, Nafion 211 membrane were purchased from Fuel Cell Store. Carbon black (VXC72, 99.5%) was supplied by Cabot. All of these reagents were used as received without any purification process. Carbon-based gas diffusion layers (GDLs, AvCarb GDS3250) were purchased from Xima Laya Photo-Electric Technology Co., Ltd., China. 99.999% purity CO_2 and Ar were both purchased from Air Liquide. Ultrapure water (18.25 MΩ·cm) supplied by a UP Water Purification System was used as the solvent throughout the experiments.

Catalyst synthesis

For the synthesis of Ag@C, monodisperse SiO_2 particles were prepared as templates through the Stöber method and redispersed in absolute ethanol at 2-3 wt% SiO_2 concentration.[1] Then, 16 mL of freshly prepared $[Ag(NH_3)_2]^+$ ions solution (0.24 mol L^{-1}) was quickly added into 18 mL of SiO_2 dispersion with stirring at room temperature for 1 h. The dispersion was added into 80 mL of ethanol containing 1.6 g of PVP and stirred at 70 ℃ for 7 h. The as-obtained SiO_2@Ag was added to a solution containing ethanol (30 mL), deionized water (5 mL) and ammonia aqueous solution (3 mL) under stirring after centrifugal washing. Then, 0.2 g of resorcinol and 0.28 mL of formaldehyde solution were added and the solution was kept stirring for 24 h at room temperature. Subsequently, the solution was transferred to an autoclave (40 mL) and hydrothermally treated at 100 ℃ for 12 h to obtain SiO_2@Ag@RF. SiO_2@Ag@C was prepared by annealing SiO_2@Ag@RF at 800 ℃ for 3 h in an Ar atmosphere. Ag@C

was finally obtained by immersing the sample in NaOH solution (5 mol L^{-1}) to etch SiO_2.[2] C@Ag was prepared through a synthetic process similar to that of Ag@C catalyst. After the obtainment of SiO_2 templates, SiO_2@RF was synthesized directly through the modified Stöber coating method, in which formaldehyde and resorcinol were used as precursors. Then, SiO_2@C was prepared through the same annealing procedure in an Ar atmosphere. The as-obtained SiO_2@C was mixed with freshly prepared $[Ag(NH_3)_2]^+$ ions solution and subsequently reduced to SiO_2@C@Ag by PVP in ethanol solution. After the removal of SiO_2 in NaOH solution, the C@Ag catalyst was finally synthesized. For the synthesis of Ag/C, 0.2 g of carbon black and 0.22 g of $AgNO_3$ were added to 60 mL deionized water and kept stirring for 4 h. The composites were dried in a baking oven at 80 ℃ for 24 h and then annealed at 300℃ for 3 h in air to obtain Ag/C.

Electrode preparation

To prepare gas diffusion electrode, 21 mg of catalyst (Ag@C or Ag/C), 2 mL of deionized water, 2 mL of isopropanol and 32 μL of FAA solution (10 wt%) were mixed under sonication for 1 h to obtain a homogeneous catalyst ink. Then, the as-prepared ink was deposited onto a 3×3 cm^2 carbon paper through the airbrushing method with the catalyst loading on GDEs of about 1 mg cm^{-2}. The GDEs was dried at room temperature overnight for further electrochemical measurements.

Electrochemical measurements

All electrochemical CO_2 reduction measurements were performed in a typical three-electrode flow cell reactor connected to an electrochemical workstation (Autolab PGSTAT204). The effective electrode geometric area is 1 cm^2 for both cathode and anode. For the CO_2RR in alkaline conditions, 1.0 M KOH was used as the electrolyte. Anolyte and catholyte chambers were separated by an anion exchange membrane (FAA-3-50). Hg/HgO was used as reference electrode and the potentials were converted to the RHE reference scale using the relation: E (vs RHE) = E (vs Hg/HgO) + 0.098 V + 0.0591 × pH. For acidic CO_2RR, 0.05 M H_2SO_4 and 0.5 M K_2SO_4 (pH 1.1) were

employed as the electrolyte. The anolyte and catholyte chambers were separated by Nafion 211 membrane. The potentials were measured *versus* the Hg/Hg_2SO_4 reference electrode and converted to the RHE scale using the relation: E (vs RHE) = E (vs Hg/Hg_2SO_4) + 0.656 V + 0.0591 × pH. For both systems, IrO_x/Ti was used as the counter electrode and the flow rate of the electrolytes were set to ~15 mL min^{-1} by Peristaltic pumps (EC200-01, Gaossunion Co., Ltd.). A mass flow controller (MC-Series, Alicat Scientific) was used to set the CO_2 gas flow rate at 20 sccm.

Product analysis

The gas products were analyzed by online gas chromatography (GC7890B, Agilent Technologies, Inc.) equipped with a thermal conductivity detector (TCD) connected to a MolSieve 5A packed column for the detection of H_2, O_2, N_2 and a back flame ionization detector (FID) connected to a Porapak Q packed column for the detection of CO. Ar was employed as the carrier gas. The Faradic efficiency (FE) of each product was calculated as follows:

$$FE = V \times C \times N \times F / (I \times V_m) \times 100\% \quad (1)$$

Where V is the gas flow rate, C is the concentration of single product in ppm, N is the electron transfer for every detected product molecule, F is the Faradic constant (F = 96485 C mol^{-1}), I is the total current and V_m is the unit molar volume of the outlet gas.

The single pass carbon efficiency (SPCE) of CO_2 towards CO was calculated based on the following equation at 25 ℃, 1 atm:

$$SPCE = j \times 24.05 / (N \times F \times V) \times 100\% \quad (2)$$

Where j is the partial current density of CO, N is the electron transfer for the generation of one CO molecule, V is the inlet flow rate of CO_2.

Mass transport simulations

The concentration of local species (H^+, OH^-, CO_2, HCO_3^-, CO_3^{2-}) were tracked by finite-element method (FEM) simulations based on a reaction-diffusion model. A sector domain was selected as the model for the calculation. The ring with a thickness of 40

nm represented the carbon layer and a liquid diffusion layer was assumed to be 50 μm as suggested by previous publications.[3-5]

In simulations, the following electrochemical reactions (CO_2RR and HER) on the inner surface of catalysts and homogenous equilibrium reactions in the entire domain were considered.

$$CO_2 + H_2O + 2e^- \rightarrow CO + 2OH^- \quad (3)$$

$$2H_2O + 2e^- \rightarrow H_2 + 2OH^- \quad (4)$$

$$CO_2 + H_2O \rightleftharpoons H^+ + HCO_3^- \quad (5)$$

$$HCO_3^- \rightleftharpoons H^+ + CO_3^{2-} \quad (6)$$

$$H_2O \rightleftharpoons H^+ + OH^- \quad (7)$$

$$CO_2 + OH^- \rightleftharpoons HCO_3^- \quad (8)$$

$$HCO_3^- + OH^- \rightleftharpoons H_2O + CO_3^{2-} \quad (9)$$

The transport of relevant species is based on the following equations:

$$\frac{\partial c_i}{\partial t} + \nabla j_i = R_i \quad (10)$$

$$j_i = -D_{e,i}\frac{\partial c_i}{\partial x} \quad (11)$$

Where j_i is the molar flux, R_i is the rate of each species which can be broken into electrochemical reactions and carbonate equilibria reactions. $D_{e.i}$ is the effective diffusivity in the carbon layer, which is corrected with porosity, ε_m, and tortuosity, τ_m, of the medium using the Bruggeman relationship according to related work.[6] The species diffusion coefficients are listed in Table S3.

$$D_{e.i} = \frac{\varepsilon_m}{\tau_m}D_i = {\varepsilon_m}^{3/2}D_i \quad (12)$$

The ε_m is calculated according to the definition:

$$\varepsilon_m = \frac{V_p}{V_0} \quad (13)$$

Where V_P is the volume of pores, V_0 is the volume of the catalyst carbon layer. According to BET analysis (Figure S4), V_P is set to be 0.18m cm^3, where m is the mass of catalyst. V_0 is calculated as follows:

$$V_0 = \frac{m}{\rho} \times \frac{\frac{4}{3}\pi(R^3 - r^3)}{\frac{4}{3}\pi R^3} \quad (14)$$

Where ρ is the density of the catalyst, R is the radius of the spherical catalyst,

r is the radius of the cavity. The density of Ag@C catalyst was estimated by the drainage method. Both R and r can be obtained from TEM images. Based on this method, the porosity of the catalyst carbon layer is calculated to be 0.3.

The rate of electrochemical reactions is calculated as follows:

$$r_{CO_2} = -\frac{i}{F}\frac{FE_{CO}}{2} \quad (15)$$

$$r_{OH^-} = -\frac{i}{F} \quad (16)$$

In the model, a constant supply of CO_2 was set at the outer surface of the carbon layer and the maximum concentration of CO_2 is calculated based on Henry's Law.

$$C^0_{co2,aq} = K^0_H C^0_{co2,gas} \quad (17)$$

$$\ln(K^0_H) = 93.4517\left(\frac{100}{T}\right) - 60.2409 + 23.3585 ln\frac{T}{100} \quad (18)$$

Where K^0_H is the Henry's constant, T is the temperature and T=293.15 K. The saturated concentration of CO_2 is further corrected due to the high concentration of ions in the electrolyte by using Sechenov equation.

$$\log\left(\frac{C^0_{co_2,aq}}{C_{co_2,aq}}\right) = K_s C_s \quad (19)$$

$$K_s = \sum(h_{ion} + h_G) \quad (20)$$

$$h_G = h_{G,0} + h_T(T - 298.15) \quad (21)$$

Where C_s is the molar concentration, K_s is the Sechenov's constant which is calculated based on previous publications.[7, 8]

Supplementary Figures

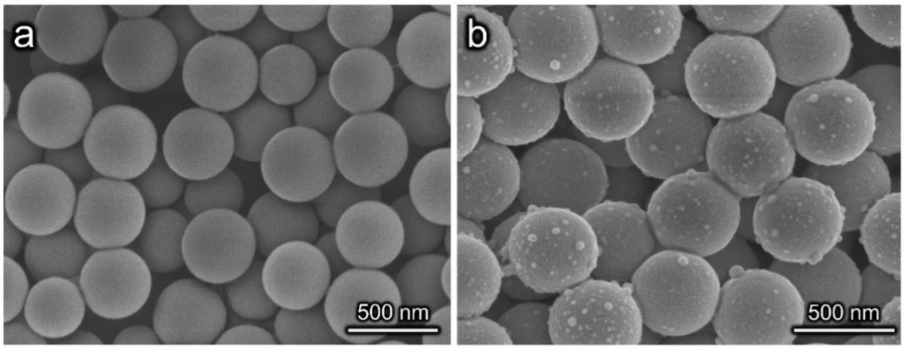

Figure S1. SEM images of (a) SiO_2 and (b) SiO_2@Ag.

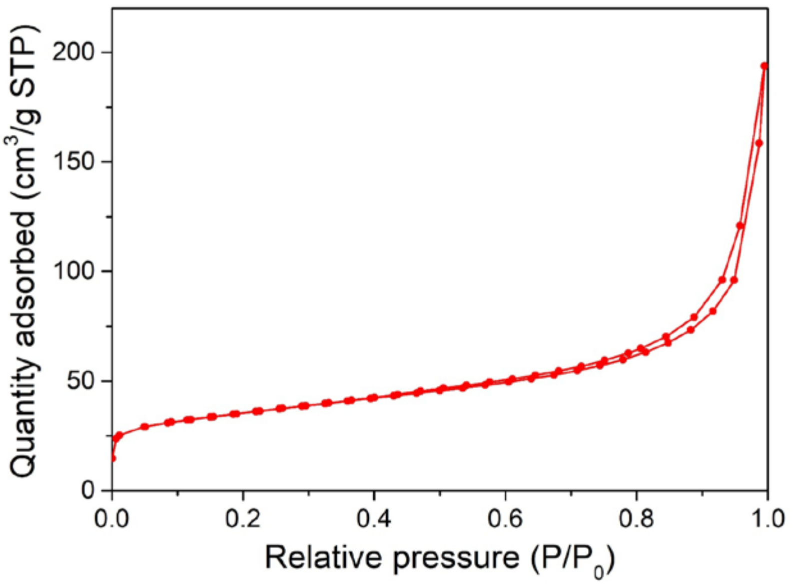

Figure S2. N_2 adsorption-desorption isotherm of Ag@C catalyst.

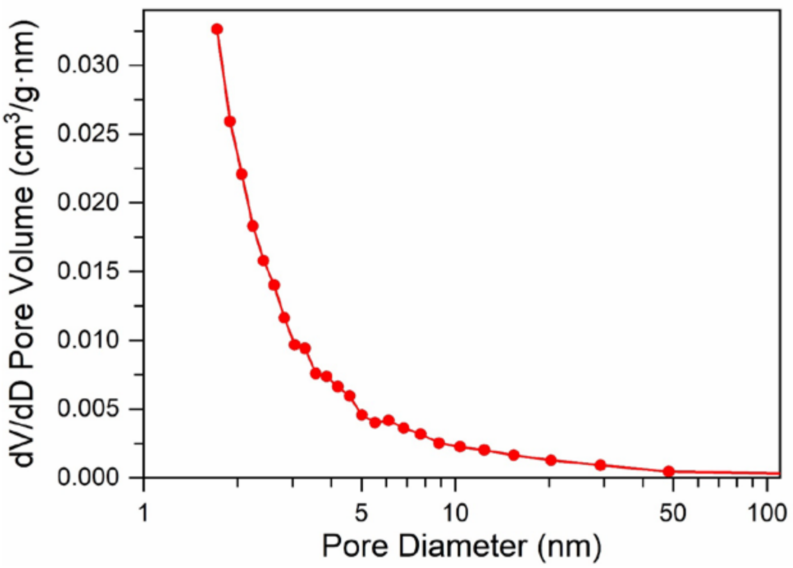

Figure S3. Pore size distribution of Ag@C catalyst.

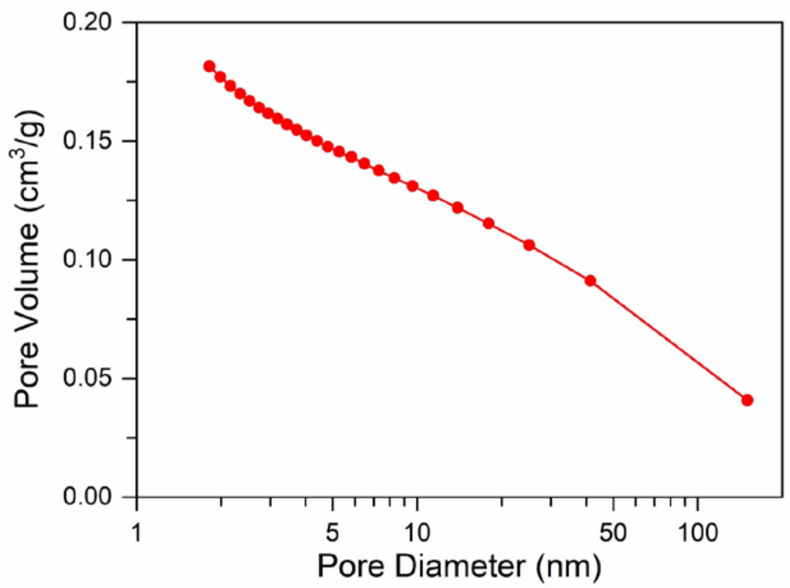

Figure S4. Cumulative pore volume of Ag@C catalyst.

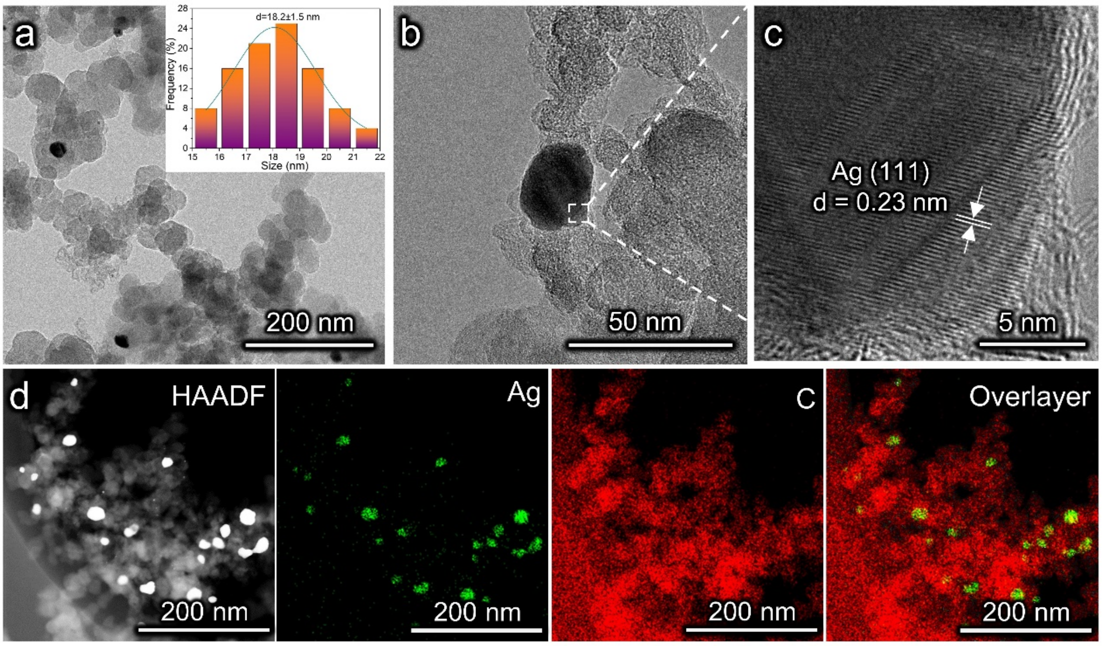

Figure S5. (a, b) TEM, (c) HRTEM and (d) EDS elemental mapping images of the Ag/C catalyst. The inset in (a) is the size distribution of metal particles.

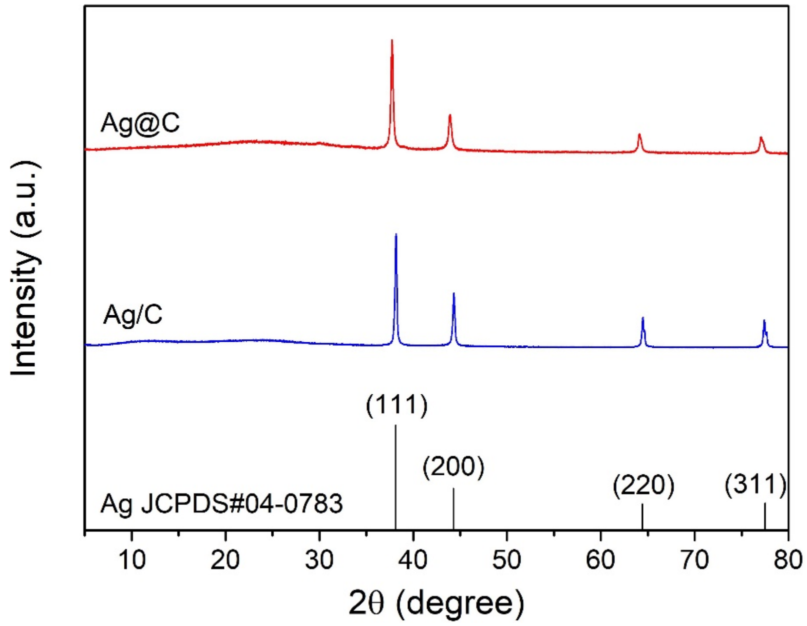

Figure S6. XRD patterns of the Ag@C and Ag/C catalysts.

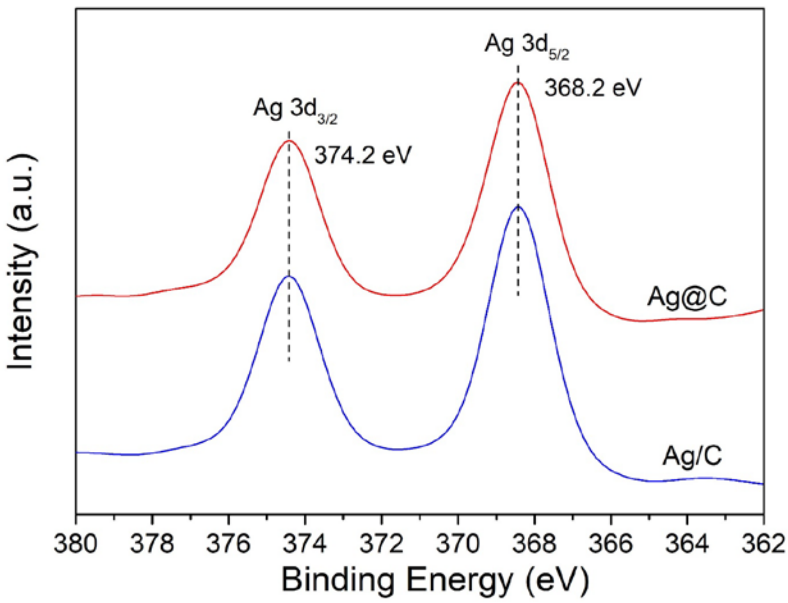

Figure S7. XPS spectra of the Ag@C and Ag/C catalysts.

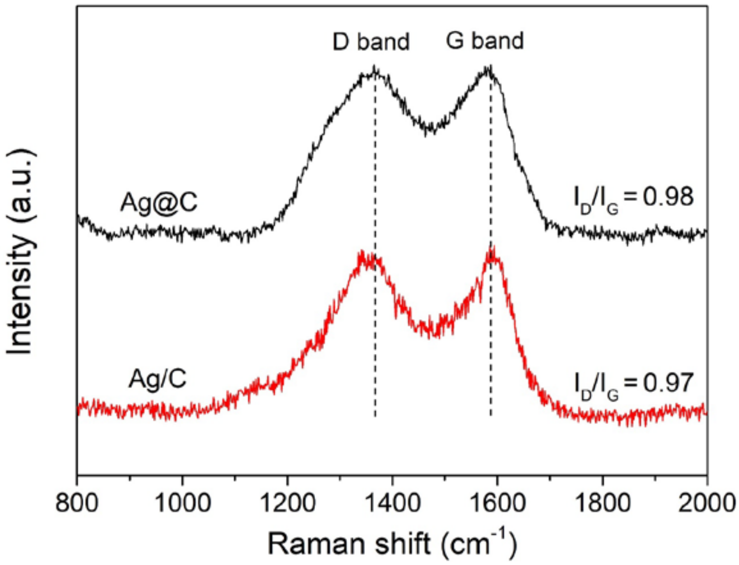

Figure S8. Raman spectra of the Ag@C and Ag/C catalysts. The ratios of peak intensity (I_D/I_G) are similar.

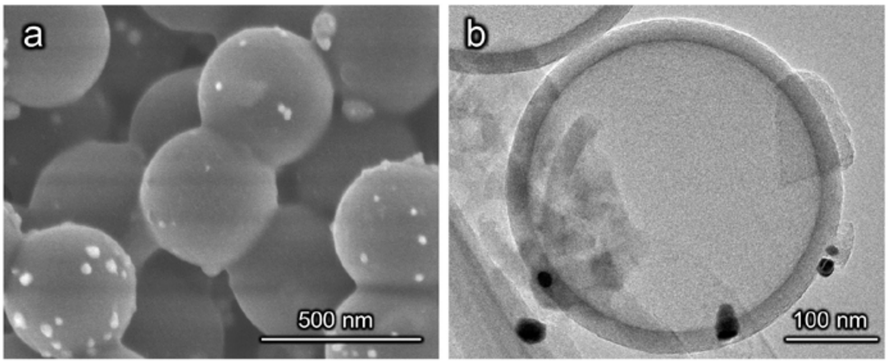

Figure S9. (a) SEM and (b) TEM images of C@Ag catalyst.

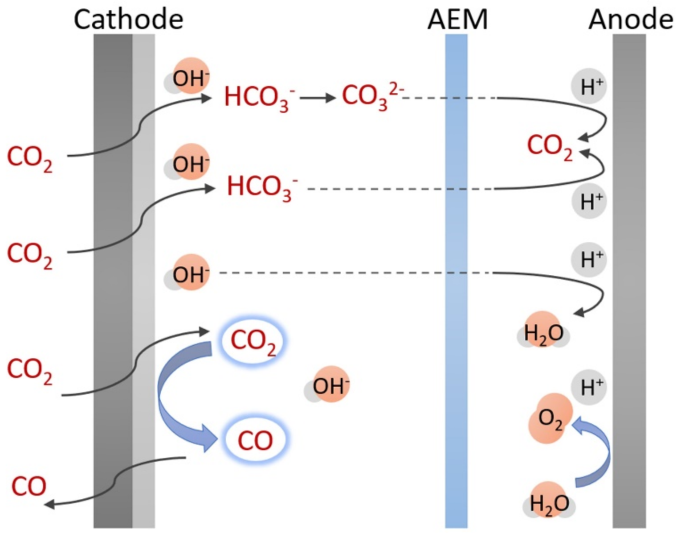

Figure S10. Schematic of carbonate formation and crossover in alkaline media.

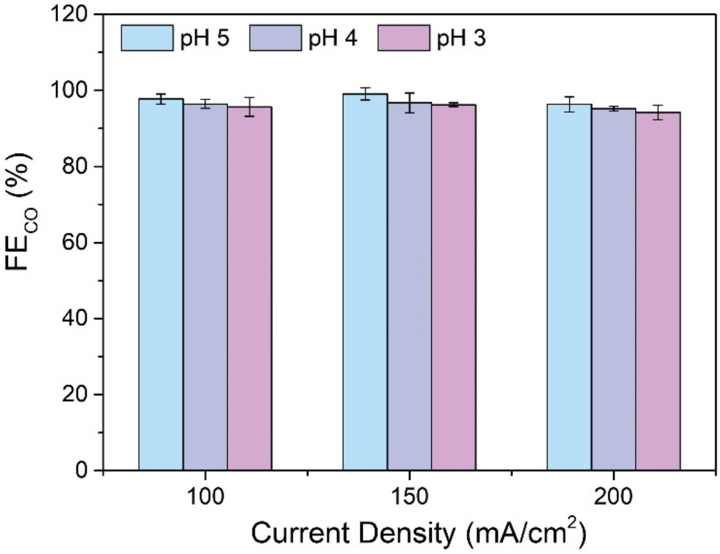

Figure S11. CO_2 electroreduction performance of Ag@C in 0.5 M K_2SO_4 electrolyte with different pH obtained by the adjustment with H_2SO_4.

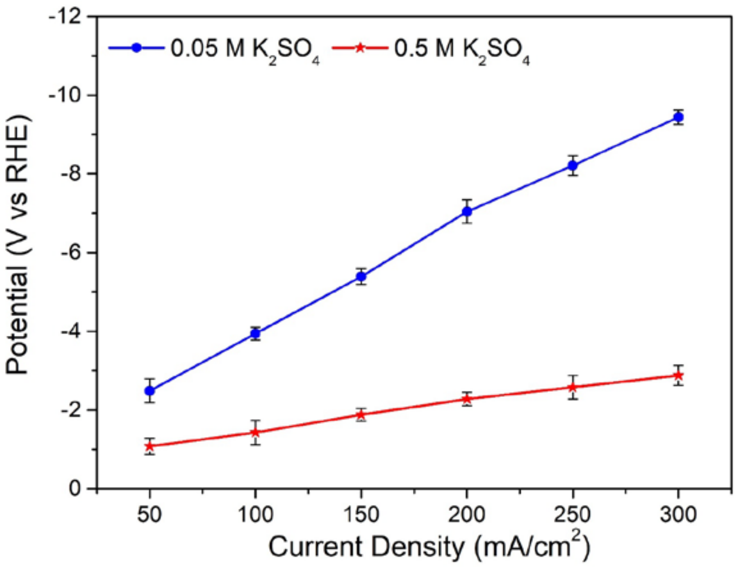

Figure S12. Cathodic potentials in 0.5 M K_2SO_4 and 0.05 M K_2SO_4 (pH 4.0).

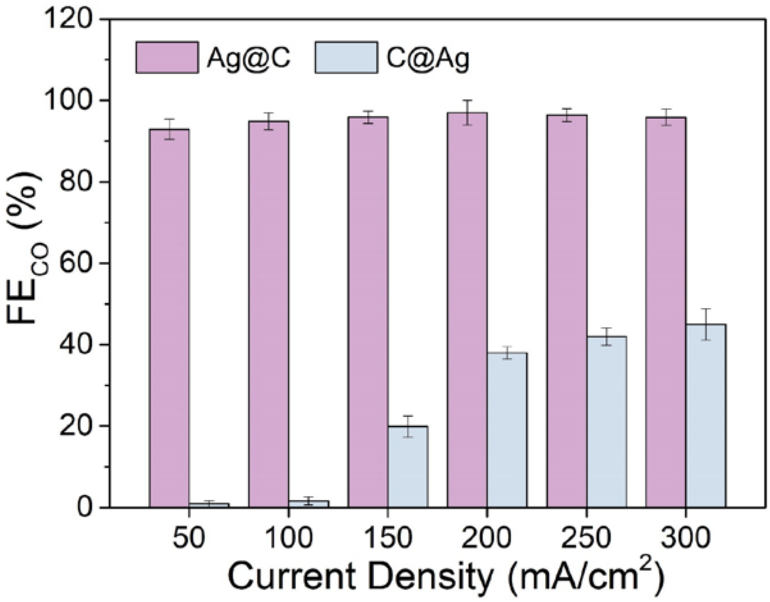

Figure S13. FE_{CO} of Ag@C and C@Ag catalysts at different current densities in 0.5 M K_2SO_4 (pH 1.1).

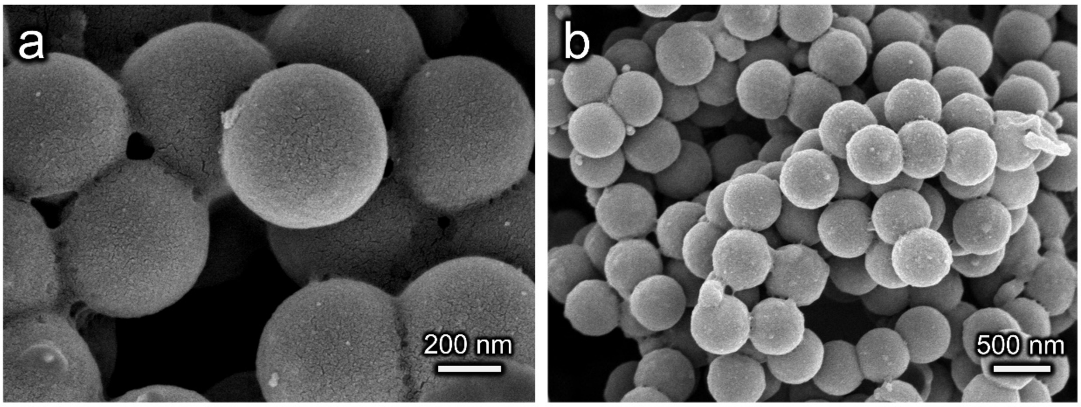

Figure S14. SEM images of Ag@C after CO_2RR stability test in 0.5 M K_2SO_4 (pH 1.1).

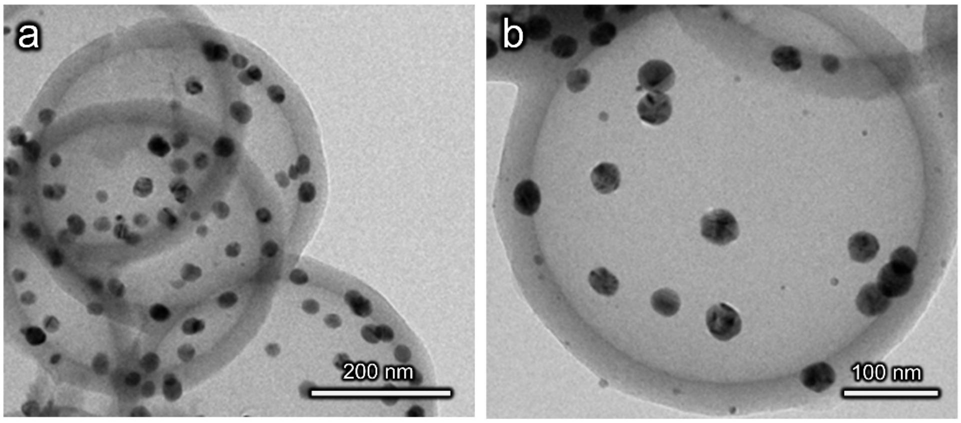

Figure S15. TEM images of Ag@C after CO_2RR stability test in 0.5 M K_2SO_4 (pH 1.1).

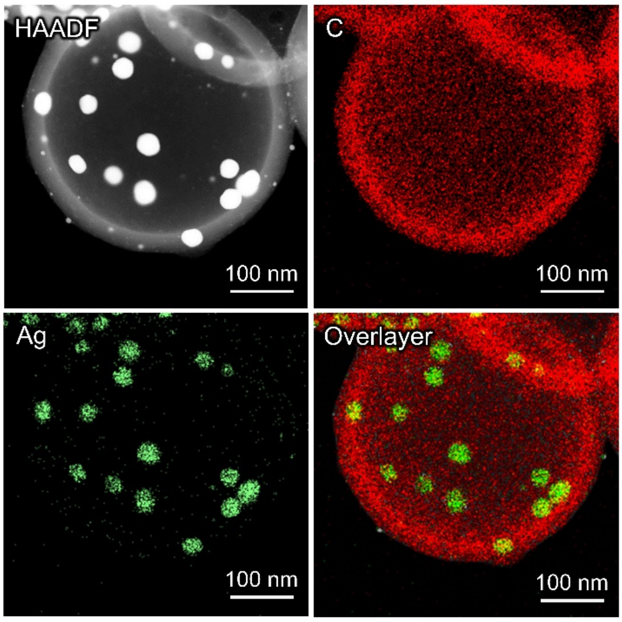

Figure S16. EDS elemental mapping images of Ag@C after CO_2RR stability test in 0.5 M K_2SO_4 (pH 1.1).

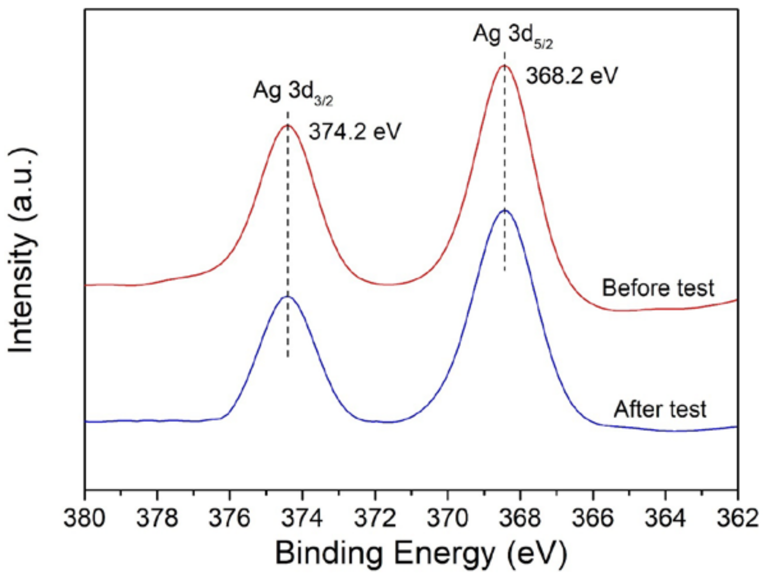

Figure S17. XPS spectra of Ag@C catalyst before and after CO_2RR stability test in 0.5 M K_2SO_4 (pH 1.1).

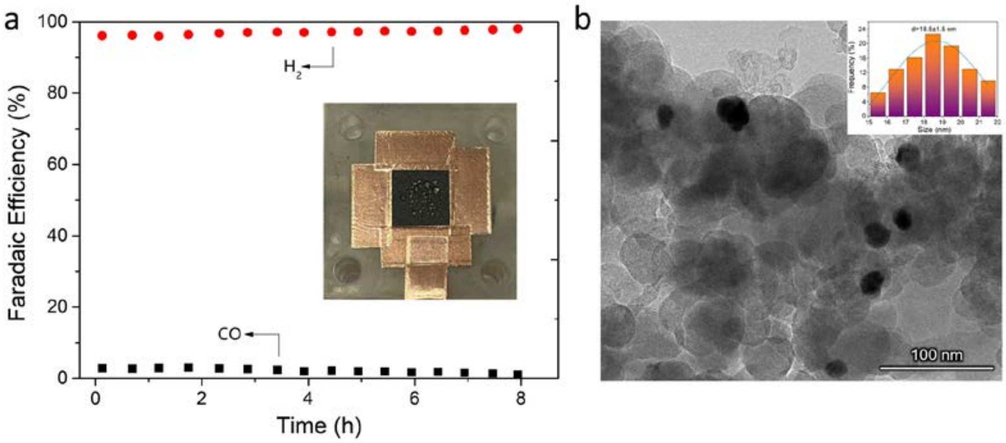

Figure S18. (a) Stability performance of Ag/C catalyst at 100 mA cm^{-2} in 0.5 M K_2SO_4 (pH 1.1). Inset shows the photograph of the backside of GDE after stability test. (b) TEM image of Ag/C after CO_2RR stability test. Inset is the particle size distribution of Ag.

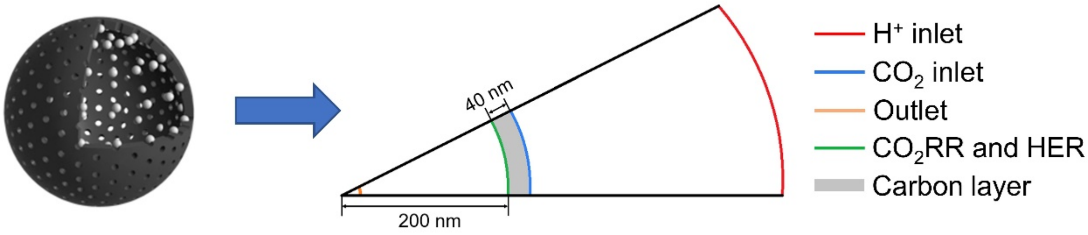

Figure S19. Graphical illustration of the model.

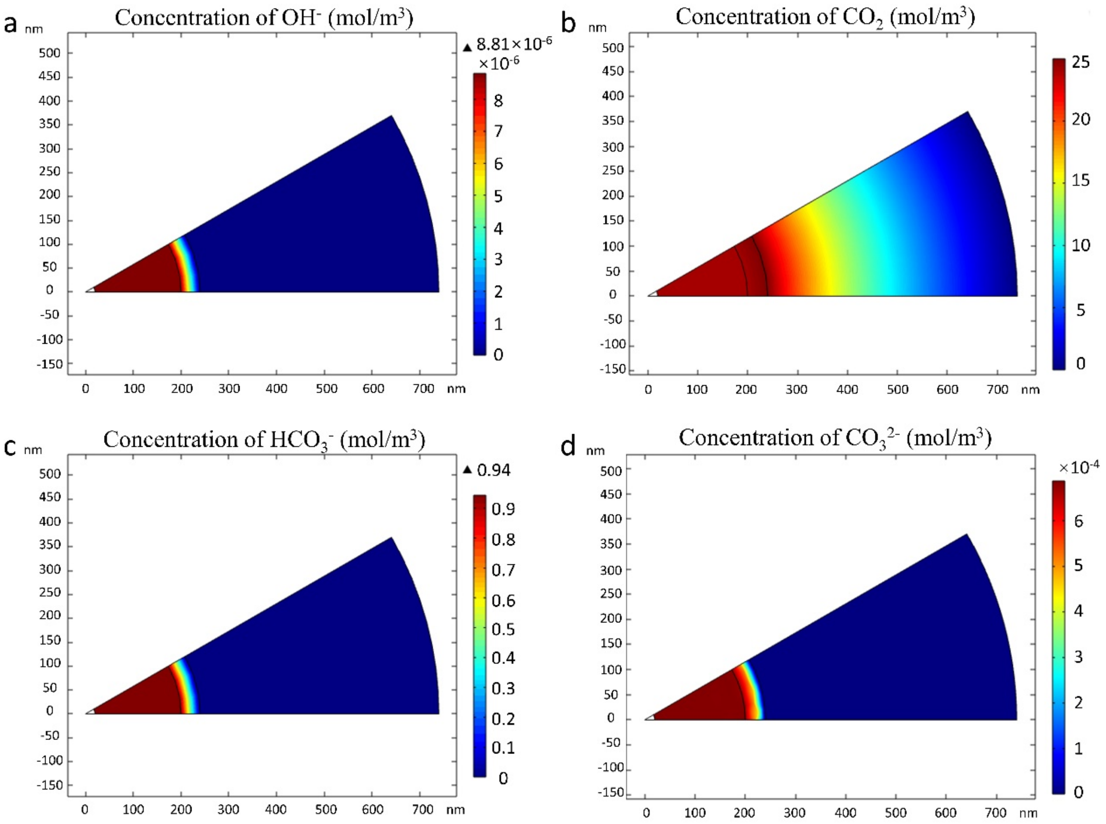

Figure S20. Computed concentration distribution of species at 50 mA cm^{-2} (pH 1.1). (a) OH^-. (b) CO_2. (c) HCO_3^-. (d) CO_3^{2-}.

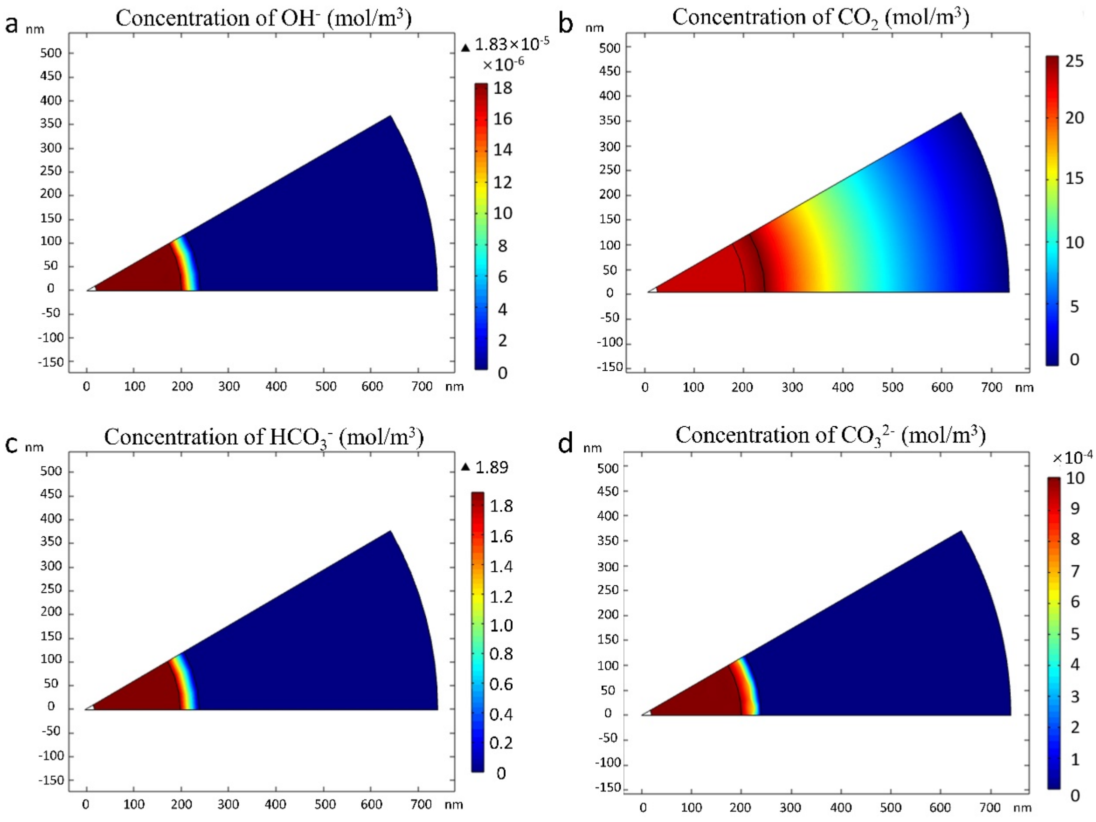

Figure S21. Computed concentration distribution of species at 100 mA cm^{-2} (pH 1.1). (a) OH^-. (b) CO_2. (c) HCO_3^-. (d) CO_3^{2-}.

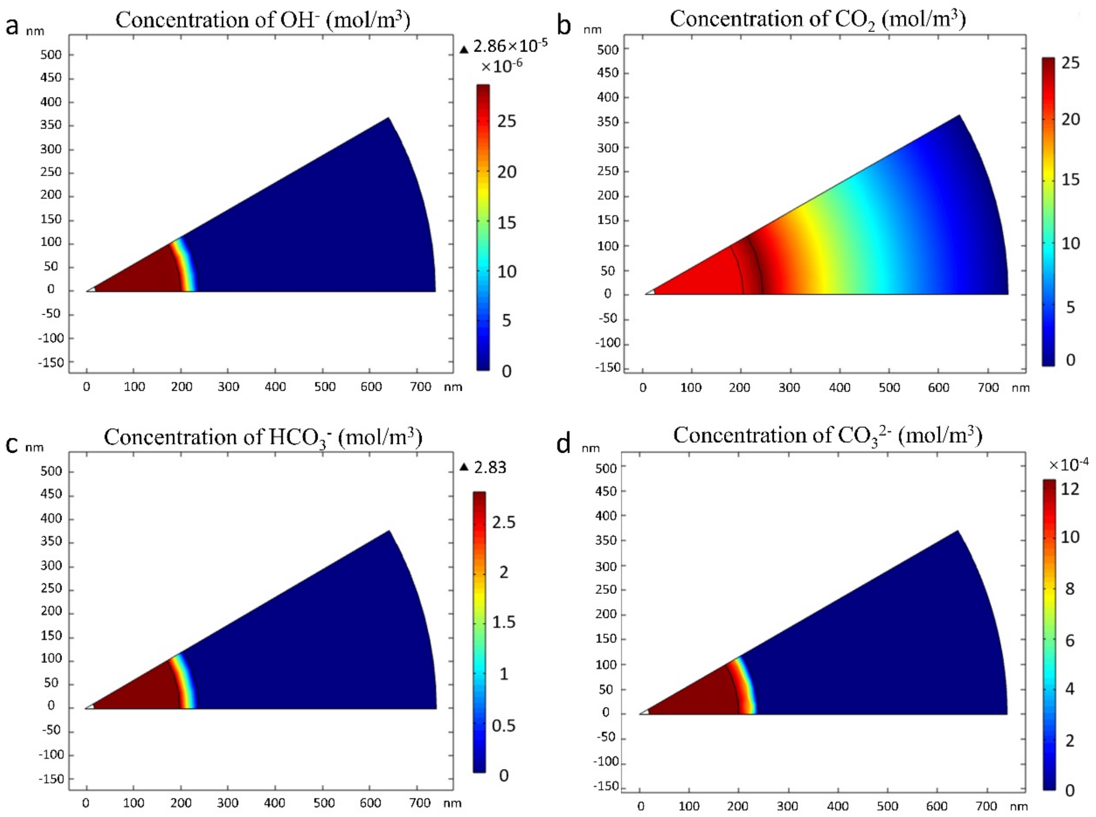

Figure S22. Computed concentration distribution of species at 150 mA cm^{-2} (pH 1.1). (a) OH^-. (b) CO_2. (c) HCO_3^-. (d) CO_3^{2-}.

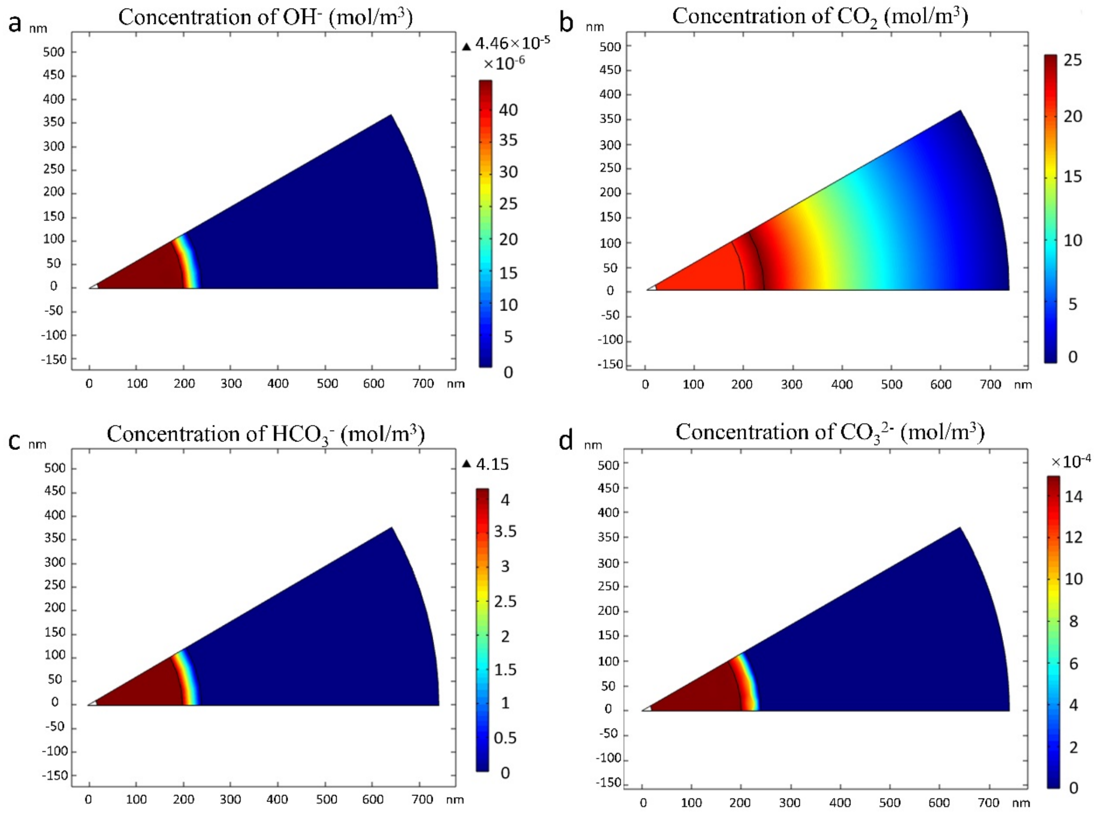

Figure S23. Computed concentration distribution of species at 200 mA cm^{-2} (pH 1.1). (a) OH^{-}. (b) CO_2. (c) HCO_3^{-}. (d) CO_3^{2-}.

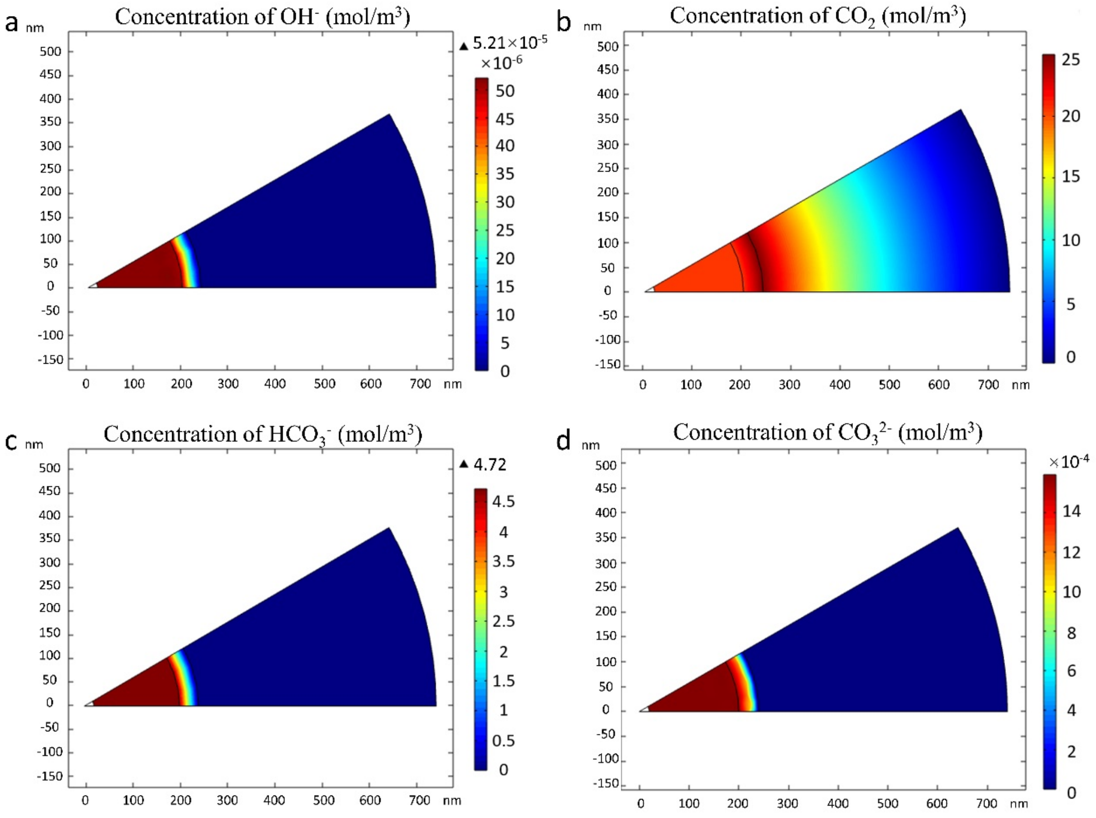

Figure S24. Computed concentration distribution of species at 250 mA cm^{-2} (pH 1.1). (a) OH^{-}. (b) CO_2. (c) HCO_3^{-}. (d) CO_3^{2-}.

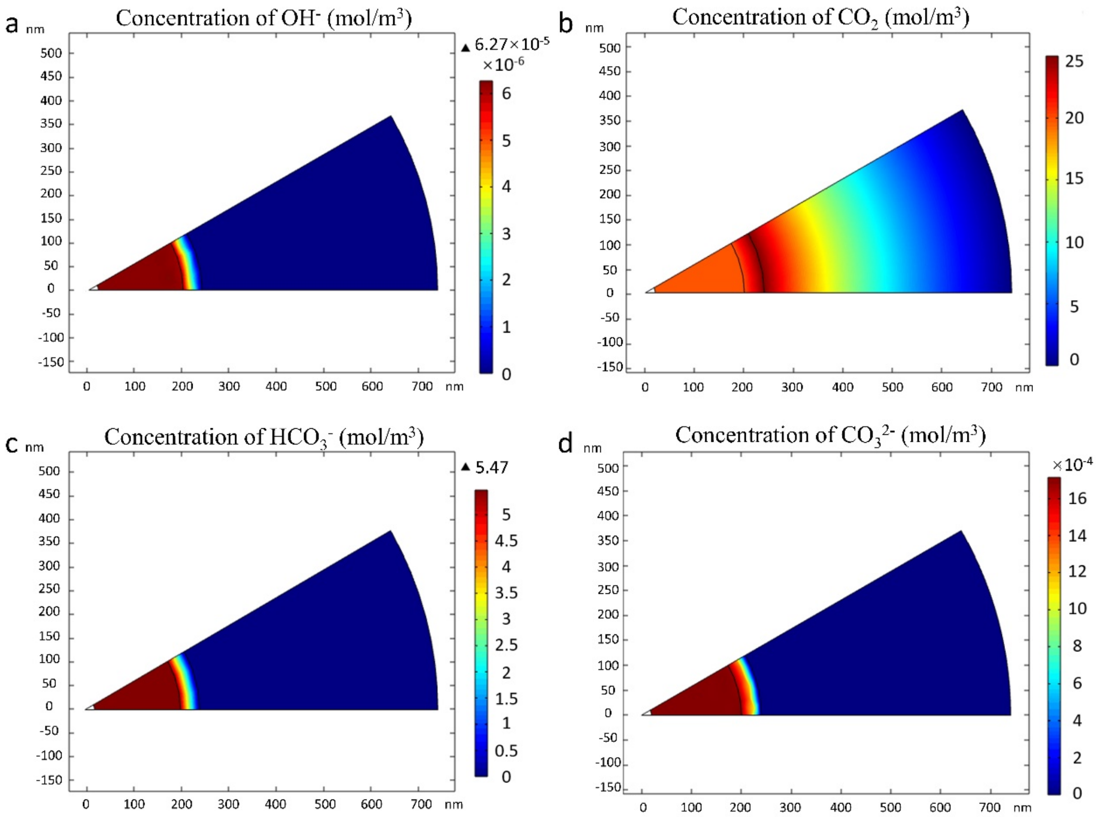

Figure S25. Computed concentration distribution of species at 300 mA cm^{-2} (pH 1.1). (a) OH^-. (b) CO_2. (c) HCO_3^-. (d) CO_3^{2-}.

Table S1. Composition of Ag@C and Ag/C catalysts measured by ICP-OES

Sample	Ag content (wt%)	C content (wt%)
Ag@C	38.8	61.2
Ag/C	42.4	57.6

Table S2. The amount of dissolved Ag in the electrolyte quantified by ICP-OES

Sample	Dissolved Ag
Before stability test	No detected
After stability test	No detected

Table S3. Diffusion coefficients

Diffusion coefficient	Value (10^{-9} m^2 s^{-1})
D_{H^+}	9.310
D_{OH^-}	5.273
D_{CO_2}	1.910
$D_{HCO_3^-}$	1.185
$D_{CO_3^{2-}}$	0.923

References

1. Z. W. Deng, M. Chen and L. M. Wu, *J. Phys. Chem. C*, 2007, **111**, 11692-11698.
2. S. Feng, W. Li, Q. Shi, Y. Li, J. Chen, Y. Ling, A. M. Asiri and D. Zhao, *Chem. Commun.*, 2014, **50**, 329-331.
3. N. Gupta, M. Gattrell and B. MacDougall, *J. Appl. Electrochem.*, 2005, **36**, 161-172.
4. Y. C. Tan, K. B. Lee, H. Song and J. Oh, *Joule*, 2020, **4**, 1104-1120.
5. J. E. Huang, F. W. Li, A. Ozden, A. S. Rasouli, F. P. G. de Arquer, S. J. Liu, S. Z. Zhang, M. C. Luo, X. Wang, Y. W. Lum, Y. Xu, K. Bertens, R. K. Miao, C. T. Dinh, D. Sinton and E. H. Sargent, *Science*, 2021, **372**, 1074-1078.
6. L. C. Weng, A. T. Bell and A. Z. Weber, Phys. Chem. Chem. Phys., 2018, **20**, 16973-16984.
7. Y. Xie, P. Ou, X. Wang, Z. Xu, Y. C. Li, Z. Wang, J. E. Huang, J. Wicks, C. McCallum, N. Wang, Y. Wang, T. Chen, B. T. W. Lo, D. Sinton, J. C. Yu, Y. Wang and E. H. Sargent, *Nat. Catal.*, 2022, **5**, 564-570.
8. Y. Qiao, W. Lai, K. Huang, T. Yu, Q. Wang, L. Gao, Z. Yang, Z. Ma, T. Sun, M. Liu, C. Lian and H. Huang, *ACS Catal.*, 2022, **12**, 2357-2364.
